# Three new species of the spider genus *Luzonacera* Li & Li, 2017 from Philippines (Araneae, Psilodercidae)

**DOI:** 10.3897/zookeys.822.30927

**Published:** 2019-02-04

**Authors:** Wan-Jin Chang, Fengyuan Li, Shuqiang Li

**Affiliations:** 1 Institute of Zoology, Chinese Academy of Sciences, Beijing 100101, China Institute of Zoology, Chinese Academy of Sciences Beijing China; 2 University of Chinese Academy of Sciences, Beijing, China University of Chinese Academy of Sciences Beijing China

**Keywords:** cave, endemic, Luzon Island, Southeast Asia, tropical

## Abstract

Three new species of *Luzonacera* Li & Li, 2017 are described: *L.francescoballarini* Li & Li, **sp. n.** (♂♀), *L.lattuensis* Li & Li, **sp. n.** (♂♀) and *L.peterjaegeri* Li & Li, **sp. n.** (♂♀). Prior to this study, the genus was known by two species, both from Luzon Island, Philippines. So far, the genus and all five species are endemic to Luzon Island and can be found in dry or humid caves in a dark environment.

## Introduction

The spider family Psilodercidae Machado, 1951 contains eleven genera and 116 species (World Spider Catalog 2018, Li and Quan 2017). All species are restricted to tropical Asia and known from Sri Lanka and India to Philippines (World Spider Catalog 2018). Currently, five species of Psilodercidae belonging to four genera are known to occur in Philippines (World Spider Catalog 2018): *Psilodercesegeria* Simon, 1892 from Luzon, *Althepusnoonadanae* Brignoli, 1973 from Mindanao, *Leclerceranegros* Deeleman-Reinhold, 1995 from Negros, and *Luzonacerachang* Li & Li, 2017 and *L.duan* Li & Li, 2017 from Luzon.

The recently described genus *Luzonacera* Li & Li, 2017 was known from two species, *L.chang* Li & Li, 2017 and *L.duan* Li & Li, 2017 (World Spider Catalog 2018). While studying new material collected on Luzon Island, we recognized three new species of the genus. The goal of this paper is to provide detailed descriptions of these new species.

## Materials and methods

All specimens were collected in Luzon Island and preserved in 95% ethanol solution. All types are deposited in the Institute of Zoology, Chinese Academy of Sciences in Beijing (IZCAS) and Senckenberg Research Institute in Frankfurt (SMF). A Leica M205 C stereomicroscope was used to measure and examine the specimens. Morphological details of the specimens were studied with an Olympus BX41 compound microscope. An Olympus C7070 wide zoom digital camera (7.1 megapixels) mounted on an Olympus SZX12 stereomicroscope was used to take photos. The images were generated using Helicon Focus 6.7.1 image stacking software and further revised with Adobe Photoshop. Leg measurements are shown as total length (femur, patella, tibia, metatarsus, and tarsus). Leg segments were measured from their retrolateral side except for *L.peterjaegeri* sp. n. which was measured from the prolateral side. All measurements are given in millimetres (mm). Terminology follows that of Li et al. (2014), Tong and Li (2007) and Deeleman-Reinhold (1995).

The extraction of genomic DNA from legs followed Li and Li (2018). Primer sets for the PCR and cycle sequencing reactions used for cytochrome c oxidase subunit I (COI) in this study are from Folmer et al. (1994). All sequences were analysed using BLAST. The GenBank accession numbers are provided in Table 1. The COI dataset of the three sequences obtained in this study and two sequences from GenBank were aligned using MAFFT version 7 (http://mafft.cbrc.jp/ alignment/server/). MEGA7.0.16 (Kumar et al. 2016) was used for subsequent manual adjustment of the sequences and calculation of pairwise comparisons of uncorrected K2P-distances.

**Table 1.** The accession numbers for each species in this paper.

Species	Length (bp)	GenBank accession number
*Luzonacerafrancescoballarini* sp. n.	651	MK238752
*Luzonaceralattuensis* sp. n.	651	MK238753
*Luzonacerapeterjaegeri* sp. n.	651	MK238754

## Taxonomy

### Family Psilodercidae Machado, 1951

#### Genus Luzonacera

Taxon classificationAnimaliaAraneaeOchyroceratidae

Li & Li, 2017

##### Type species.

*Luzonacerachang* Li & Li, 2017

##### Emended diagnosis.

*Luzonacera* resembles *Althepus* Thorell, 1898 and *Leclercera* Deeleman-Reinhold, 1995. However, *Luzonacera* can be differentiated by the combination of the following characteristics: 1) absence of a conductor (versus presence of a conductor in both *Althepus* and *Leclercera*); 2) absence of a retrolateral protrusion on the tibia or cymbium of the male palp (versus presence of a retrolateral protrusion on the tibia or cymbium of the male palp in *Althepus* and *Leclercera*); 3) remarkably inflated tibia of the male palp; 4) pyriform bulb with spirally extended embolus; and 5) two pairs of spermathecae, the lateral spermathecae with longer stalks than the medial spermathecae.

##### Composition.

*L.chang* Li & Li, 2017 (the type species), *L.duan* Li & Li, 2017, *L.francescoballarini* sp. n., *L.lattuensis* sp. n. and *L.peterjaegeri* sp. n.

##### Distribution.

Philippines.

#### Illustrated key to the males of *Luzonacera*

**Table:** 

1	Embolus and bulb equal in length; embolus and bulb ratio approximately equal to 1 (Fig. 1i)	*** L. chang ***
–	Embolus short (bulb ca. 2 times longer than the embolus); embolus and bulb ratio: 0.40–0.57 (Fig. 1ii–v)	**2**
2	Slight constriction of the central part of bulb (Fig. 1iii)	***L.francescoballarini* sp. n.**
–	Pronounced constriction of the central part of bulb (Fig. 1ii, iv, v)	**3**
3	Bulb with smooth surface dorsally (Fig. 1v)	***L.peterjaegeri* sp. n.**
–	Bulb with a notch (NO) dorsally (Fig. 1ii & iv)	**4**
4	The tip of the bulb without protrusion (PT) (Fig. 1ii)	*** L. duan ***
–	The tip of the bulb with protrusion (PT) (Fig. 1iv)	***L.lattuensis* sp. n.**

**Figure 1.**
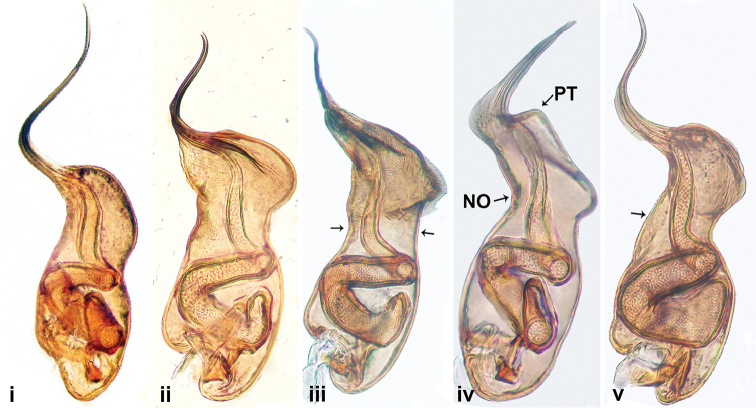
Prolateral view of male palp bulbs of *Luzonacera* species: **i***L.chang***ii***L.duan***iii***L.francescoballarini* sp. n. **iv***L.lattuensis* sp. n. and **v***L.peterjaegeri* sp. n. Abbreviations: **PT** = protrusion, **NO** = notch.

#### Illustrated key to the females of *Luzonacera*

**Table:** 

1	Two pairs of similar spermathecae (Fig. 2a–d)	**2**
–	Two pairs of dissimilar spermathecae (medial pair oblique, tube-shaped) (Fig. 2e)	***L.peterjaegeri* sp. n.**
2	Spermathecae without globose distal part (with swollen distal ends) (Fig. 2a)	*** L. chang ***
–	Spermathecae with globose distal part (Fig. 2b–d)	**3**
3	Two pairs of spermathecae pointed almost the same direction (Fig. 2b, d)	**4**
–	Two pairs of spermathecae pointed opposite directions (Fig. 2c)	***L.francescoballarini* sp. n.**
4	Relatively short stalks of medial spermathecae; distal part and stalk ratio approximately 0.5 (Fig. 2d)	***L.lattuensis* sp. n.**
–	Relatively long stalks of medial spermathecae; distal part and stalk ratio approximately 0.25 (Fig. 2b)	*** L. duan ***

**Figure 2.**
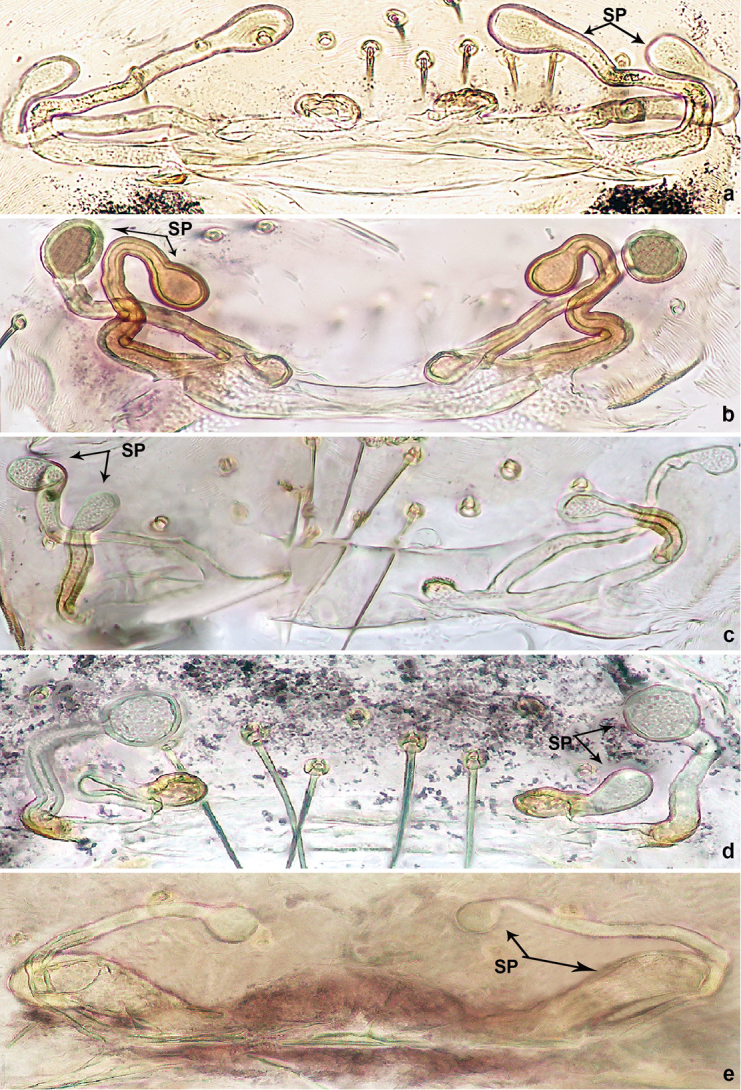
Female internal genitalia of five different species of *Luzonacera*: **a***L.chang***b***L.duan***c***L.francescoballarini* sp. n. **d***L.lattuensis* sp. n. and **e***L.peterjaegeri* sp. n. Abbreviation: **SP** = spermathecae.

##### Luzonacera francescoballarini

Taxon classificationAnimaliaAraneaeOchyroceratidae

Li & Li sp. n.

http://zoobank.org/EEE59EF0-7FF6-4F7D-BB5E-6FE76729FB76

Figs 3 , 4 , 9 , 10

###### Types.

**Holotype**: ♂ (IZCAS), Philippines, Luzon Island, Bulacan Province, San Miguel City, near Biak-Na-Bato National Park, Bayukbok Cave, 15°10'5.4"N, 121°5'4.3"E, 125 m, 21.V.2015, F. Ballarin and Y. Li. **Paratypes**: 1♂, 1♀ (IZCAS), same data as holotype.

###### Etymology.

The species is named after Francesco Ballarin, who collected the type series; name in genitive case.

###### Diagnosis.

**Figure 3.**
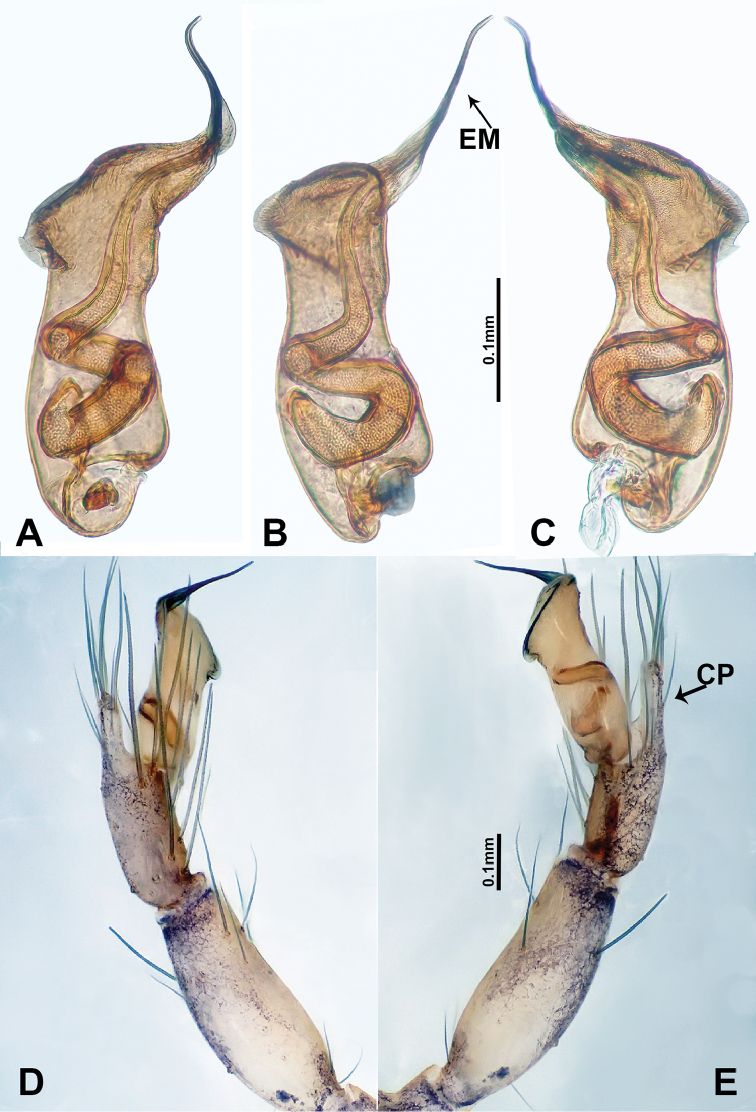
*Luzonacerafrancescoballarini* sp. n., male holotype **A** palp bulb, retrolateral view **B** palp bulb, ventral view **C** palp bulb, prolateral view **D** palp, prolateral view **E** palp, retrolateral view. Abbreviations: **EM** = embolus; **CP** = cymbial protrusion.

**Figure 4.**
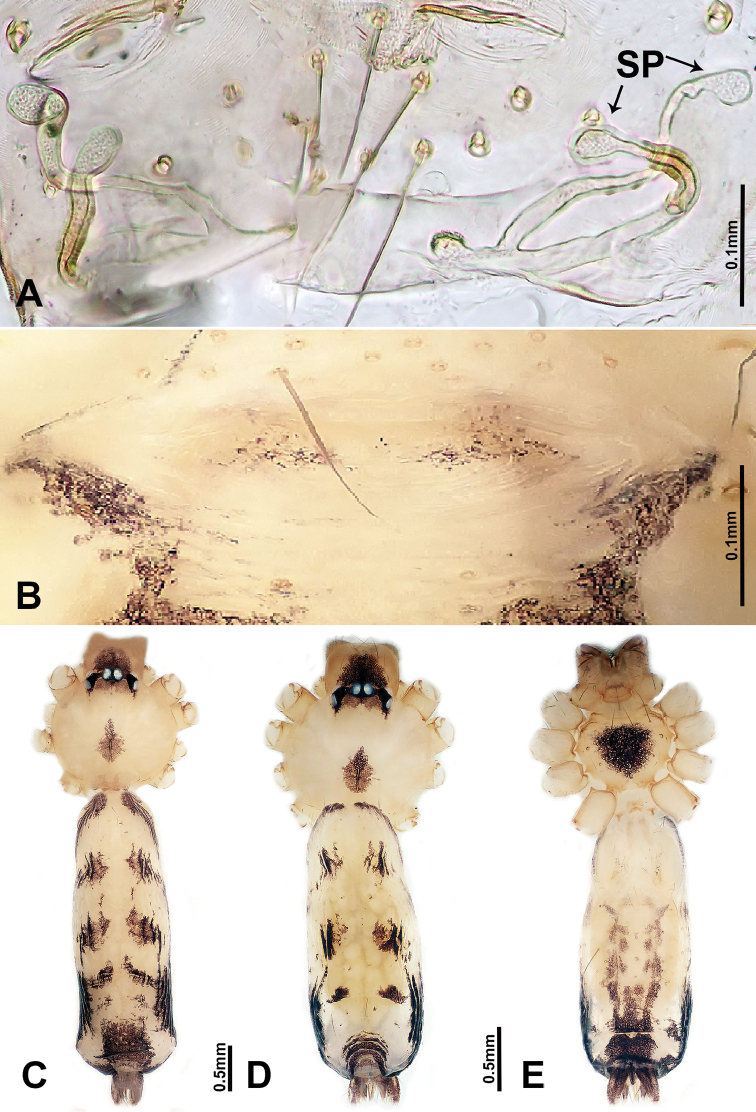
*Luzonacerafrancescoballarini* sp. n., male holotype and female paratype **A** internal genitalia, dorsal view **B** female epigastric furrow, ventral view **C** male habitus, dorsal view **D** female habitus, dorsal view **E** female habitus, ventral view. Abbreviation: **SP** = spermathecae.

*Luzonacerafrancescoballarini* sp. n. resembles *L.lattuensis* sp. n. in having a short embolus, and two pairs of twisted spermathecae globose at distal parts. Males can be distinguished from the latter species by the smooth dorsal surface of the bulb (Figure 3B); females can be distinguished by having longer spermathecae (Figure 4A; versus shorter in *L.lattuensis* sp. n. in Figure 4A).

###### Description.

**Male** (Holotype). Total length 4.81; carapace 1.60 long, 1.28 wide; abdomen 3.20 long, 0.96 wide. Colour faded. Carapace round, pale yellow, with ovoid brown patch medially and rounded brown patch posterior to ocular area. Fovea shallow. Anterior margin of thoracic region distinctly elevated. Chelicerae light brown with lamina, promargin with a single tooth and retromargin with two small teeth (Figure 9A). Clypeus slanting, light brown. Labium slanting, pale brown. Sternum pale brown with large patch of brown spots medially. Abdomen elongated with complex patterns dorsally and ventrally. Legs light brown; measurements: I 22.44 (6.73, 0.64, 6.41, 3.21, 5.45), II 17.63 (5.13, 0.64, 5.13, 5.45, 1.28), III 12.05 (3.53, 0.32, 3.40, 3.20, 1.60), IV 23.07 (7.05, 0.64, 7.05, 6.41, 1.92). Palp (Figure 3A–E): tibia swollen at the base, length/width = 2.40; cymbium swollen with distal protrusion and numerous long setae; length/width = 2.33; bulb light brown, pyriform; embolus forms a slender spiral extending from tip of bulb.

**Female** (paratype). Similar to male in coloration and general features but slightly larger (Figure 4D, E). Measurements: total length 3.59; carapace 1.20 long, 1.40 wide; abdomen 2.20 long, 0.80 wide. Leg measurements: I 14.11(4.17, 0.32, 4.17, 4.17, 1.28), II 10.88 (3.50, 0.32, 3.53, 3.21, 0.32), III 9.26 (2.56, 0.50, 2.60, 2.60, 1.00), IV 14.06 (4.49, 0.40, 4.17, 4.00, 1.00). Internal genitalia: two pairs of slender spermathecae with long stalks (ca. 6 times longer than distal globular parts), spermathecae distal parts not wider than basal width of stalks, both pairs equal in width (Figure 4A).

###### Distribution.

Type locality only (Figure 10).

###### Natural history.

Collected in a dark and rather humid cave, close to the ground, along the wall of the cave with huge rocks.

###### Comments.

Based on the 651 bp aligned sequences, the COI uncorrected K2P-distance between *L.francescoballarini* sp. n. and *L.chang* is 13.5%, between *L.francescoballarini* sp. n. and *L.duan* is 15.0%, between *L.francescoballarini* sp. n. and *L.lattuensis* sp. n. is 14.9%, and between *L.francescoballarini* sp. n. and *L.peterjaegeri* sp. n. is 13.9%.

##### Luzonacera lattuensis

Taxon classificationAnimaliaAraneaeOchyroceratidae

Li & Li sp. n.

http://zoobank.org/0D355C5B-450C-4F82-805E-0062E14381F0

Figs 5 , 6 , 9 , 10

###### Types.

**Holotype**: ♂ (IZCAS), Philippines, Luzon Island, Cagayon Province, Tuguegarao City, Penablanca Village, Lattu-Lattuc Cave, 17°42'23"N, 121°49'2"E, 111 m, 31.V.2015, F. Ballarin and Y. Li. **Paratypes**: 1♂, 1♀ (IZCAS), same data as holotype.

###### Etymology.

The species name is an adjective referring to the type locality.

###### Diagnosis.

**Figure 5.**
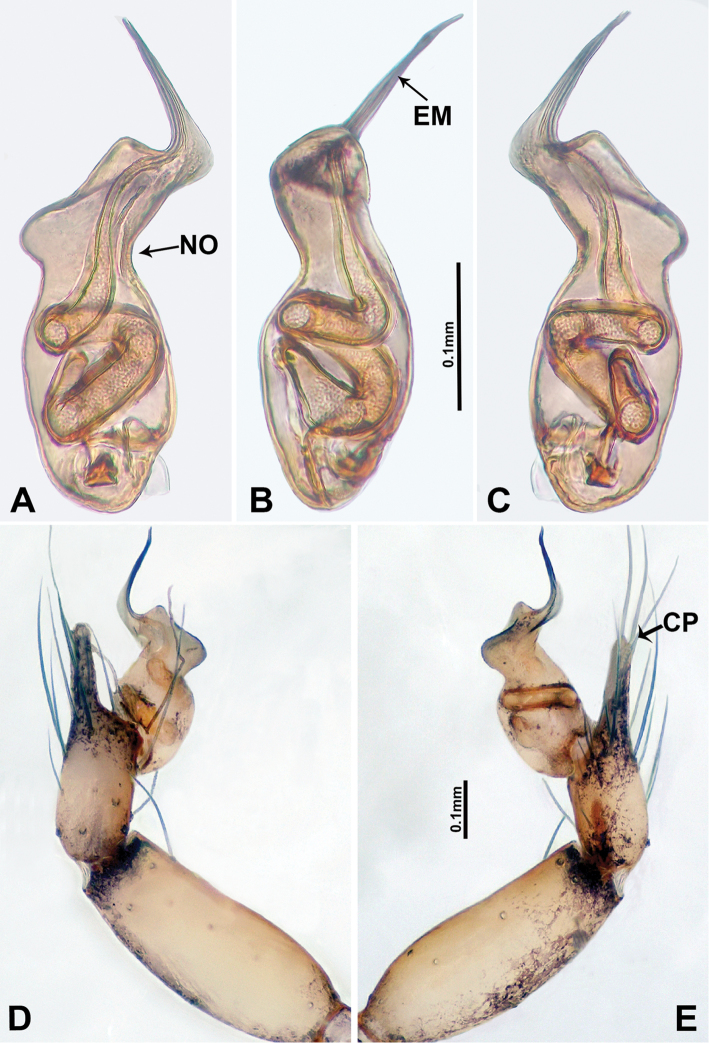
*Luzonaceralattuensis* sp. n., male holotype **A** palp bulb, retrolateral view **B** palp bulb, ventral view **C** palp bulb, prolateral view **D** palp, prolateral view **E** palp, retrolateral view. Abbreviations: **EM** = embolus; **CP** = cymbial protrusion; **NO** = notch.

**Figure 6.**
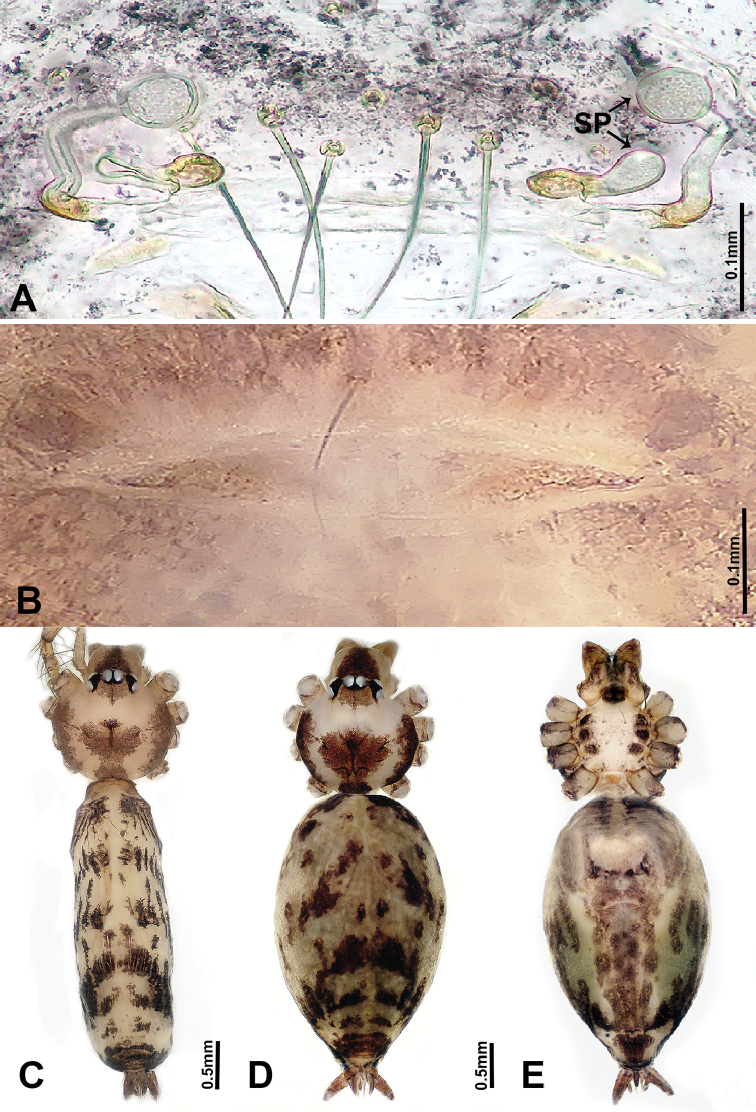
*Luzonaceralattuensis* sp. n., male holotype and female paratype **A** internal genitalia, dorsal view **B** female epigastric furrow, ventral view **C** male habitus, dorsal view **D** female habitus, dorsal view **E** female habitus, ventral view. Abbreviation: **SP** = spermathecae.

Both sexes of *L.lattuensis* sp. n. and *L.francescoballarini* sp. n. are very similar. Males of *L.lattuensis* sp. n. can be distinguished from *L.francescoballarini* sp. n. by the bulb with a dorsal notch (Figure 5A) and a relatively longer cymbium tip (Figure 5E); females can be distinguished by having shorter spermathecae with more widely spaced bases (Figure 6A; versus longer spermathecae with more narrowly spaced bases in *L.francescoballarini* sp. n. in Figure 8A).

###### Description.

**Male** (Holotype). Total length 3.85; carapace 1.28 long, 0.96 wide; abdomen 2.56 long, 0.75 wide. Carapace round and brown, with three longitudinal brown bands; the central band is 3 times wider than the lateral bands (Figure 6C). Fovea shallow, brown. Anterior margin of thoracic region distinctly elevated. Chelicerae brown with lamina, promargin with one tooth and retromargin with two small teeth (Figure 9B). Clypeus slanting, brown with two pale rounded areas laterally and two triangular projections basally. Labium slanting, dark brown. Sternum pale brown with three dark brown patches laterally. Abdomen elongated, with complex patterns dorsally and ventrally. Legs brown with white annulations; measurements: I & II missing, III 7.80 (2.24, 0.32, 2.24, 2.00, 1.00), IV missing. Palp (Figure 5A–E): tibia swollen at the base, length/width = 2.50; cymbium with distal protrusion, length/width = 2.0; bulb light brown, pyriform; embolus forms a slender spiral extending subapically from bulb.

**Female** (paratype). General features and coloration are similar to the male, but the female is slightly larger (Figure 6D, E). Measurements: total length 3.81; carapace 1.25 long, 1.00 wide; abdomen 2.56 long, 1.40 wide. Leg measurements: I 11.05 (3.25, 0.40, 3.20, 3.20, 1.00), II 10.57 (3.20, 0.32, 2.88, 3.21, 0.96), III 6.74 (2.00, 0.32, 1.92, 1.75, 0.75), IV 13.35 (3.75, 0.31, 3.80, 4.49, 1.00). Internal genitalia: two pairs of twisted spermathecae, medial spermathecae with globose distal parts and short stalks, and lateral spermathecae with globose distal parts and long stalks (stalks ca. 3 times longer than distal parts) (Figure 6A).

###### Distribution.

Type locality only (Figure 10).

###### Natural history.

Collected close to the ground along the wall of a dark, rather dry and dusty secondary cave with huge rocks.

###### Comments.

Based on the 651 bp- aligned sequences, the COI uncorrected K2P-distance between *L.lattuensis* sp. n. and *L.chang* is 12.4%, between *L.lattuensis* sp. n. and *L.duan* is 11.5%, and between *L.lattuensis* sp. n. and *L.peterjaegeri* sp. n. is 13.6%.

##### Luzonacera peterjaegeri

Taxon classificationAnimaliaAraneaeOchyroceratidae

Li & Li sp. n.

http://zoobank.org/ B57BC1C9-4645-4662-90C1-CE3FFC211E96

Figs 7 , 8 , 9 , 10

###### Types.

**Holotype**: ♂ (SMF), Philippines, Northern Luzon Island, Teresita State, Cagayan Province, Lower Kimmabalyu Cave, 18°11'35.4"N, 121°52'10.3"E, 22.I.2015, H. Steiner. **Paratypes**: 1♂, 2♀ (SMF), same data as holotype.

###### Etymology.

The species is named after Peter Jäger in honour of his contribution to the study of spiders from Asia; name in genitive case.

###### Diagnosis.

**Figure 7.**
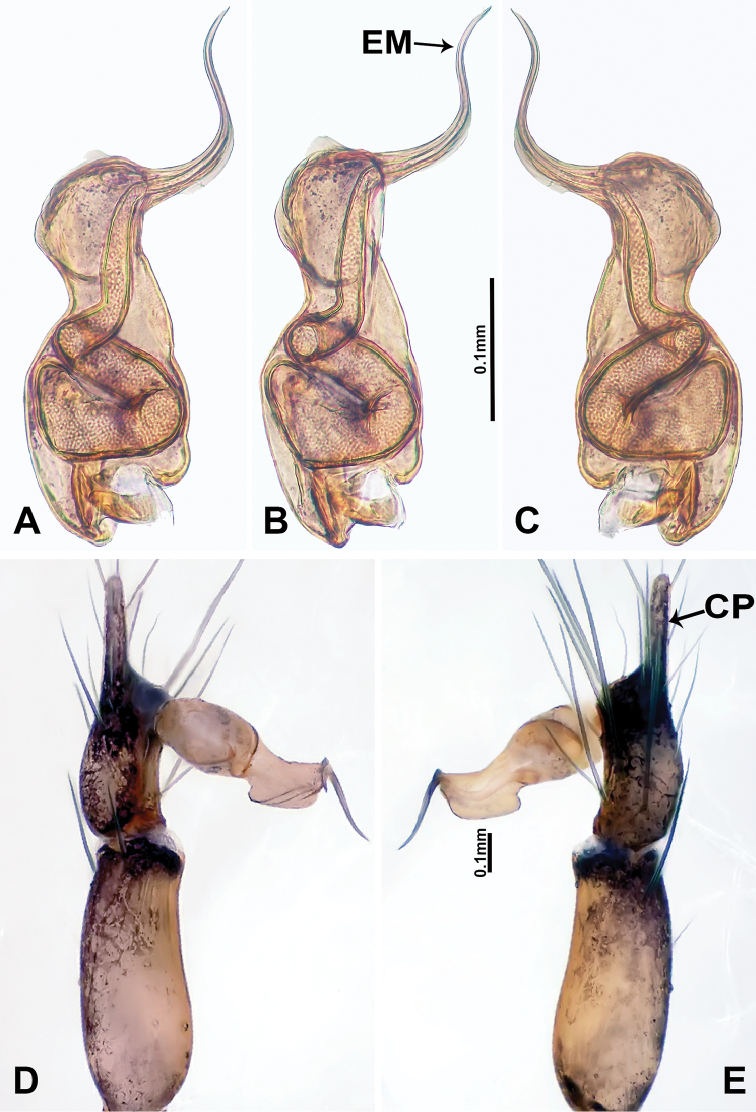
*Luzonacerapeterjaegeri* sp. n., male holotype **A** right palp bulb, retrolateral view **B** right palp bulb, ventral view **C** right palp bulb, prolateral view **D** right palp, prolateral view **E** right palp, retrolateral view. Abbreviations: **EM** = embolus; **CP** = cymbial protrusion.

**Figure 8.**
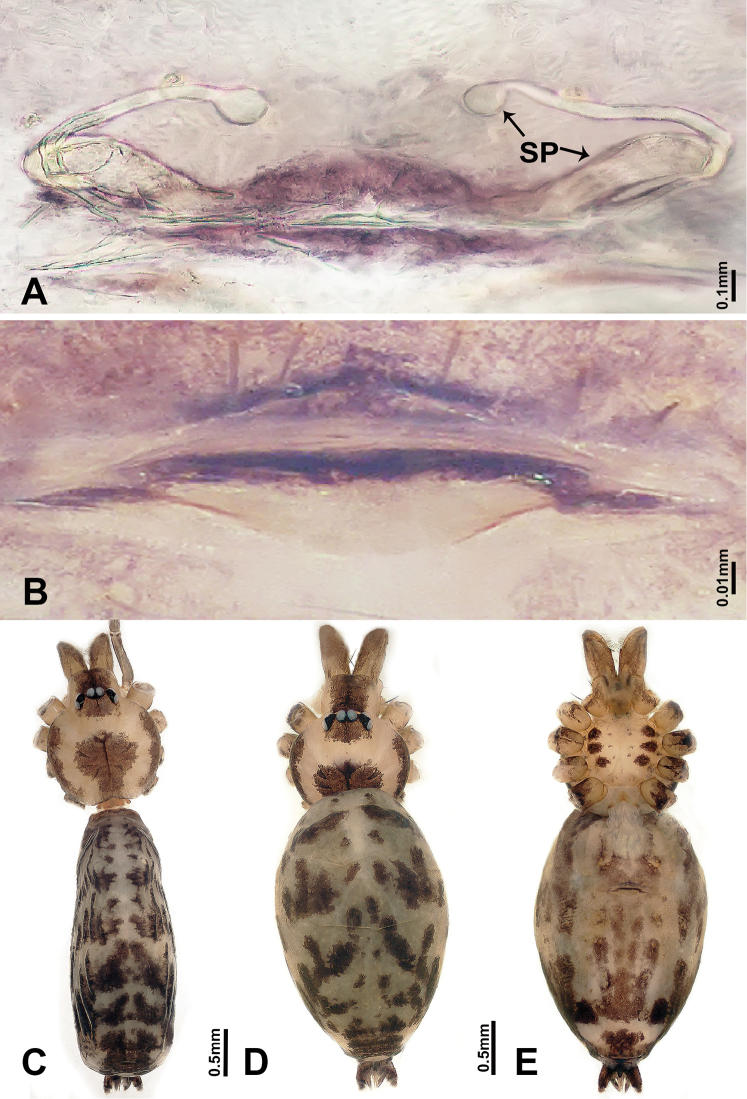
*Luzonacerapeterjaegeri* sp. n., male holotype and female paratype **A** internal genitalia, dorsal view **f** female epigastric furrow, ventral view **C** male habitus, dorsal view **D** female habitus, dorsal view **E** female habitus, ventral view. Abbreviation: **SP** = spermathecae.

*Luzonacerapeterjaegeri* sp. n. can be distinguished from all other known species of the genus by a distinct constriction on the central part of the bulb (Figure 7A); females can be distinguished by two types of spermathecae: one pair of slender spermathecae bearing a globose distal part, and one pair of oblique, tube-shaped spermathecae (Figure 8A; versus both pairs of spermathecae bearing a globose distal part in other species). Moreover, both sexes of *L.peterjaegeri* sp. n. have longer chelicerae (Figure 8; versus shorter chelicerae in other species).

###### Description.

**Figure 9.**
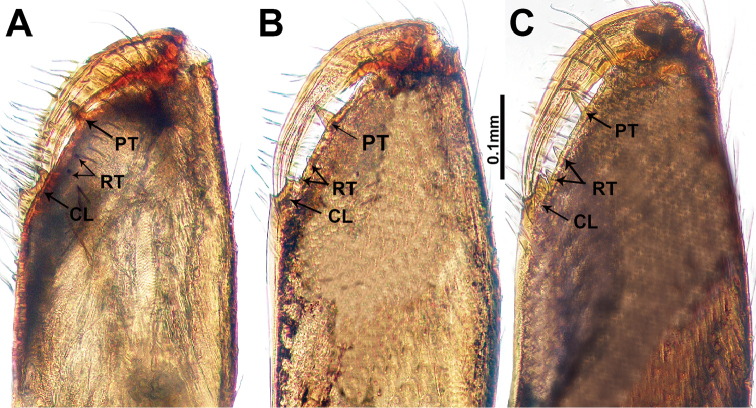
Cheliceral retromargin **A***L.francescoballarini* sp. n. **B***L.lattuensis* sp. n. **C***L.peterjaegeri* sp. n. Abbreviations: **PT** = promargin teeth; **RT** = retromargin teeth; **CL** = cheliceral lamina.

**Male** (Holotype). Total length 4.00; carapace 1.50 long, 1.40 wide; abdomen 2.50 long, 1.00 wide. Carapace round, pale brown, with three longitudinal brown bands, with the middle band 3 times wider than the lateral bands (Figure 8C). Fovea shallow and brown. Anterior margin of thoracic region distinctly elevated. Chelicerae long, brown with lamina, promargin with one tooth, and retromargin with two small teeth (Figure 9C). Clypeus brown with two pale rounded areas laterally and two relatively longer protrusions basally. Labium brown. Sternum brown with three dark brown patches laterally. Abdomen elongated, with complex patterns dorsally and ventrally. Leg measurements: all legs missing. Right palp (Figure 7A–E): tibia swollen at the base, length/width = 2.25; cymbium with distal protrusion, length/width = 3.4; bulb light brown, pyriform; embolus forms a slender spiral elongating terminally from bulb.

**Female** (paratype). General features and coloration are similar to male, but the female is slightly larger (Figure 8D, E). Measurements: total length 4.17; carapace 1.60 long, 1.28 wide; abdomen 2.56 long, 1.5 wide. Legs missing. Internal genitalia: two pairs of spermathecae, one pair of spermathecae globose distally with long stalks (ca. 6 times longer than distal parts), the other pair are oblique, tube-shaped spermathecae (Figure 8A).

###### Distribution.

**Figure 10.**
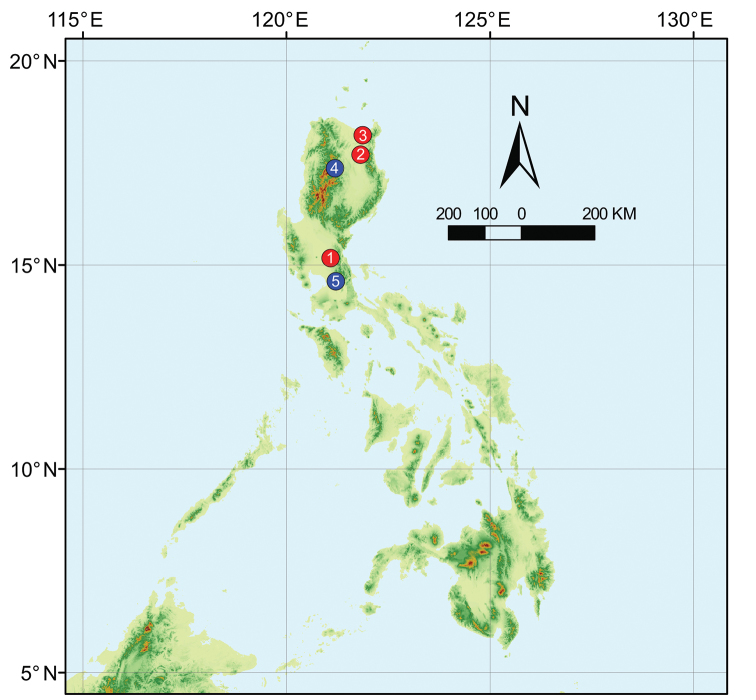
Distribution of five *Luzonacera* in Philippines **1***Luzonacerafrancescoballarini* sp. n. **2***L.lattuensis* sp. n. **3***L.peterjaegeri* sp. n. **4***L.duan***5***L.chang*.

Type locality only (Figure 10).

###### Comments.

Based on the 651 bp aligned sequences, the COI uncorrected K2P-distance between *L.peterjaegeri* sp. n. and *L.chang* is 15.9%, and between *L.peterjaegeri* sp. n. and *L.duan* is 13.9%.

## Supplementary Material

XML Treatment for Luzonacera

XML Treatment for Luzonacera francescoballarini

XML Treatment for Luzonacera lattuensis

XML Treatment for Luzonacera peterjaegeri
